# Protective Effect of Phloretin against Hydrogen Peroxide-Induced Oxidative Damage by Enhancing Autophagic Flux in DF-1 Cells

**DOI:** 10.1155/2022/8359118

**Published:** 2022-12-30

**Authors:** Dan Song, Feng Liu, Wenjing Tao, Xian Wu, Haiyang Bi, Xiangchen Li, Jianhong Shu, Dong Wang

**Affiliations:** ^1^College of Animal Science and Technology, College of Veterinary Medicine, Zhejiang A&F University; Key Laboratory of Applied Technology on Green-Eco-Healthy Animal Husbandry of Zhejiang Province, Zhejiang Provincial Engineering Laboratory for Animal Health and Internet Technology, Hangzhou 311300, China; ^2^College of Life Sciences and Medicine, Zhejiang Sci-Tech University, Hangzhou 310018, China; Shaoxing Biomedical Research Institute, Zhejiang Sci-Tech University, Shaoxing 312000, China; ^3^Institute of Animal Science, Chinese Academy of Agricultural Sciences, Beijing 100193, China

## Abstract

Phloretin (PHL) is a dihydrochalcone flavonoid isolated from the peel and root bark of apples, strawberries, and other plants with antioxidative characteristic. In this study, we aimed to investigate the protective effect and the potential mechanism of PHL on hydrogen peroxide (H_2_O_2_)-induced oxidative damage in DF-1 cells. The results showed that PHL exhibited no cytotoxic effect on DF-1 cells at concentration below 20 *μ*M. PHL markedly increased H_2_O_2_-reduced cell viability, decreased H_2_O_2_-induced apoptosis, as evidenced by reduced apoptosis rate, the upregulation of gene and protein level of Bcl-2, and the downregulation of gene and protein level of Bax and Cleaved caspase3. In addition, PHL reduced H_2_O_2_-induced reactive oxygen species (ROS) production and restored antioxidant enzymes activities as well as mitochondrial membrane potential in a dose-dependent manner. Moreover, PHL prior to H_2_O_2_ further increased LC3-II level, promoted p62 turnover and improved lysosomal function. Importantly, autophagy inhibitor chloroquine (CQ) reversed the protective effect of PHL, and increased H_2_O_2_-induced apoptosis. Furthermore, PHL inhibited the phosphorylation levels of ERK, p38, and JNK. Collectively, these results indicate that PHL could attenuate H_2_O_2_-induced oxidative injury and apoptosis by maintaining lysosomal function and promoting autophagic flux, and MAPKs pathway may be involved in this process. Our study provides evidence that PHL could as a new strategy to against oxidative damage in poultry industry.

## 1. Introduction

The modern poultry industry worldwide is constantly challenged by oxidative stress. Accumulative evidences suggested that oxidative stress is a dominant factor worsening poultry production and increasing poultry industry economic losses [1, 2]. Imbalance between reactive oxygen species (ROS) and antioxidants in cells and tissues leads to oxidative stress, thereby triggering various adverse effects, such as mitochondrial dysfunction, increased apoptosis, and cell death. Generally, as a central redox metabolite, hydrogen peroxide (H_2_O_2_) can be generated by almost all oxidative stressors (e.g., environmental heat stress, feed toxins, and microorganisms) and spreads through cell and tissues, leading to an imbalance of redox states [3]. It was reported that H_2_O_2_ exposure impaired growth performance and meat quality of broilers through NF-*κ*B signal-mediated apoptosis and abnormal autophagy [4, 5]. The redox status and aerobic metabolism of broilers breast muscle were impaired after H_2_O_2_ injection [6]. Moreover, researches showed that H_2_O_2_ induced oxidative injury and apoptosis in chicken lymphocytes [7], intestinal epithelial cells [8]. Hence, controlling oxidative stress is of great benefit to poultry production.

Autophagy is an evolutionarily conserved cellular degradation process to remove damaged or superfluous cytoplasmic components. It protects cells from damage caused by energy shortage and cytotoxicity, and promotes cell survival [9]. Dysregulated autophagy fails to clear damaged mitochondrial and cytoplasmic components, resulting in ROS overproduction and oxidative injury, which contributes to the pathogenesis of multiple human and animal pathologies [10]. Recently, autophagy has been involved in the pathophysiological process of poultry production. It was reported that autophagy reduces mitochondrial dysfunction by regulating oxidative stress in Cu-treated chicken hepatocytes [11]. Dietary folic acid supplementation increased autophagy protein Beclin1, ATG5, and LC3-II/I ratio, as well as reduced p-mTOR protein expression, thereby improving semen quality and spermatogenesis of aged testis in broiler breeder roosters [12]. Collectively, autophagy plays an important role in chicken health pathophysiology, and strategies to improve autophagy may be an effective method to protect poultry from adverse factors.

In recent years, plant derived substances extracted from fruits and vegetables as antioxidants have emerged as an appropriate strategy to combat oxidative stress. Phloretin (PHL), a kind of flavonoid compound, is present in the peel and root bark of apples, strawberries, and other plants with various biological activities, such as antioxidative and anti-inflammatory characteristic, among others [13, 14]. PHL exerts its antioxidant activity by acting as a scavenger of reactive oxygen species [15]. It could upregulate Nrf2 and antioxidant enzymes activities to alleviate palmitic acid-induced oxidative stress in human umbilical vein endothelial cells [16]. A recent study has revealed the protective effect of PHL against mitochondrial dysfunction and redox imbalance in in vitro model of NAFLD by restoring damaged autophagic flux [17]. However, there is no evidence about the effect of PHL on H_2_O_2_-induced oxidative damage in DF-1 cells, and whether autophagy is involved in this process as a potential protective mechanism has not been clarified. DF-1 cells are an immortalized cell line that spread throughout the body and divided rapidly. With enhanced growth potential [18], it has been widely used as an in vitro cytotoxicity model to study the effects of environmental pollutant [19], mycotoxin [20] or cold stress [21] and other factors on poultry health.

In the current study, we aimed to explore the influence of PHL on H_2_O_2_-induced cell viability, apoptosis, redox status, and autophagy, as well as the potential regulatory mechanisms. This study would provide important evidences that PHL could against H_2_O_2_-induced DF-1 cells oxidative injury via restoration of autophagic flux.

## 2. Materials and Methods

### 2.1. Chemical Reagents and Antibodies

PHL (purity>98%) was commercially purchased from Shanghai Yuanye Bio-Technology (Shanghai, China). 2′,7′-dichlorodihydrofluorescein diacetate (DCFH-DA) fluorogenic probe, Torin2 (SML1224), and Chloroquine (CQ, C6628) were obtained from Sigma-Aldrich. Fetal bovine serum (FBS) was purchased from ExCell Biological Technology (Shanghai, China). Dulbecco's Modified Eagle Medium F-12 (DMEM/F-12), penicillin/streptomycin, Dulbecco's Phosphate-Buffered Saline (DPBS), and pancreatin were obtained from Servicebio Technology (Wuhan, China). CCK-8 assay kit, Hoechest33258, and JC-1 stain were bought from Beyotime Biotechnology (Shanghai, China). AnnexinV/propidium iodide was purchased from Solarbio Science & Technology (Beijing, China). Antioxidant capacity assay kits were obtained from Nanjing jiancheng Bioengineering Institute (Nanjing, China). Primary antibodies against LC3 (A19665) and p62 (A7758) were purchased from ABclonal. Bax (50599-2), Bcl-2 (12789-1-AP), and cleaved Caspase-3 (19677-1-AP) were from proteintech. Goat anti-rabbit IgG-HRP (A21020) and Goat anti-mouse IgG-HRP (A21010) conjugated secondary antibody were purchased from Abbkine. Other reagents were analytically pure.

### 2.2. Cell Culture and Treatment

DF-1 cells (ATCC CRL-12203) were purchased from China Infrastructure of Cell line Resources, and incubated in T-25 cell culture flask containing DMEM/F12 supplemented with 10% FBS, 1% penicillin/streptomycin at 37°C in a 5% CO_2_ incubator [19]. Subsequently, cells were treated with different concentration of PHL (0, 1, 4, 10, 20, and 50 *μ*M) for 12 h. For H_2_O_2_ and PHL cotreated group, DF-1 cells were pretreatment with PHL at different concentration for 12 h, then cotreated with 300 *μ*M H_2_O_2_ for another 3 h, and the cell morphology was observed under a light microscope.

### 2.3. Cell Viability Assay

Cell viability was measured by commercial CCK-8 assay kit following manufacturer's instructions [19]. Briefly, DF-1 cells at a density of 1 × 10^4^ cells/well into 96-well plates were pretreated with different concentrations of PHL before 300 *μ*M H_2_O_2_ treatment. Then, 10 *μ*L CCK-8 reagent was added to cell culture well for 2 h at 37°C and absorbance was measured at 450 nm using Microplate Reader (Bio Tek, USA).

### 2.4. Hoechst 33258 Staining

Hoechst 33258 staining was performed as previously described [22]. DF-1 cells were grown at a density of 6 × 10^5^ cells/mL on glass coverslips into 6-well dishes, and pretreated with different concentrations of PHL for 12 h before 300 *μ*M H_2_O_2_ treatment. After that, the treated cells were washed with PBS and fixed with 4% paraformaldehyde for 10 min at room temperature, and then cells were stained with Hoechst 33258 for 10 min in the dark conditions. The images were captured under a fluorescence microscope (Nikon Eclipse Ts2R, Tokyo, Japan).

### 2.5. Flow Cytometry

Cell apoptosis was detected with Annexin V/FITC-PI kit by flow cytometry according to instructions by the producer as previously described [23]. DF-1 cells were grown at a density of 6 × 10^5^ cells/mL on glass coverslips into 6-well dishes, the treated cells were collected and rinsed with PBS, then resuspended in binding buffer and stained with 5 *μ*L Annexin V/FITC and 5 *μ*L PI together at room temperature for 10 min without light. The cell apoptosis rate was examined by BD Accuri C6 flow cytometer following the manufacturer's instructions.

### 2.6. Real-Time Fluorescence Quantitative PCR (qRT-PCR)

qRT-PCR was performed as previously described with minor modifications [22]. In short, DF-1 cells were grown at a density of 1 × 10^6^ cells/mL into 6 cm dishes. After appropriate treatment, the cell total RNA was extracted using commercial RNAiso plus kit (Takara, Beijing, China) and cDNA was reversed by PrimeScript RT Master Mix following the manufacturer's protocol (Takara, Beijing, China). The qRT-PCR was conducted with TB Green Premix Ex Taq II (Takara, Beijing, China) on a CFX 96 PCR system instrument (Bio-Rad, Hercules, CA). The primer sequences are as follows: GAPDH: CCACTCCTCCACCTTTG (forward) and CACCACCCTGTTGCTGT (reverse); Bax: TCCTCATCGCCATGCTCAT (forward) and CCTTGGTCTGGAAGCAGAAGA (reverse); Bcl-2: ATCGTCGCCTTCTTCGAGT (forward), and GGCCTCATACTGTTGCCGTA (reverse); Caspase3: GGCTCCTGGTTTATTCAGTCTC (forward) and ATTCTGCCACTCTGCGATTT (reverse). The relative mRNA expressions were calculated using the 2^−*ΔΔ*CT^ method, and GAPDH was used as a reference gene.

### 2.7. Western Blotting

Western blotting was performed as previously described [22]. DF-1 cells were grown at a density of 6 × 10^5^ cells/mL on glass coverslips into 6-well dishes. After appropriate treatment, the cell total protein was collected and denatured at 100°C for 10 min, separated by SDS-PAGE and transferred to polyvinylidene fluoride (PVDF) membrane. The membrane was blocked for 2 h at room temperature in 5% bovine serum albumin, and then incubated with corresponding primary antibodies at 4°C overnight. After that, the membrane was washed with PBST three times and incubated with corresponding HRP-conjugated secondary antibodies for 30 min at room temperature. The protein bands were detected using chemiluminescence reagent under an imaging system and analyzed by ImageJ software.

### 2.8. ROS Detection

ROS staining was performed as previously described [22]. DF-1 cells were grown on Lab-Tek Chambered Coverglass units at a density of 2.5 × 10^5^ cells/mL and the treated cells were stained with 10 *μ*M DCFH-DA for 30 min at 37°C cell incubator and protected from light, then cells were washed with PBS and fluorescence images were observed using a confocal laser-scanning microscope (Olympus FV3000, Tokyo, Japan).

### 2.9. Mitochondrial Membrane Potential (MMP) Detection

The MMP was measured using a JC-1 staining kit as previously described with minor modifications [24]. DF-1 cells were seeded at a density of 6 × 10^5^ cells/mL into 6-well dishes, the treated cells were coincubated with JC-1 staining for 30 min at 37°C cell incubator in the dark. Then, cells were washed twice with JC-1 buffer solution for 10 min and images were observed at 490/530 nm for JC-1 monomers and 550/600 nm JC-1 aggregates under a fluorescence microscope (Nikon Eclipse Ts2R, Tokyo, Japan).

### 2.10. Lysotracker Red Staining

Lysosomal pH was determined as previously described [25]. The treated cells were incubated with 1 *μ*M Lysotracker Red dye for 30 min at 37°C cell incubator and protected from light. Then remove lysotracker Red staining solution and add fresh cell culture solution. Fluorescence images were taken using a confocal laser-scanning microscope (Olympus FV3000, Tokyo, Japan) and quantified by ImageJ software.

### 2.11. Antioxidant Parameters Detection.

DF-1 cells were grown at a density of 6 × 10^5^ cells/mL into 6-well dishes and pretreated with different concentrations of PHL for 12 h before 300 *μ*M H_2_O_2_ treatment. Then, superoxide dismutase (SOD), glutathione peroxidase (GSH-Px) and catalase (CAT) activities were detected using commercial detection kits following the manufacturer's instructions, and the absorbance were detected at 550 nm, 412 nm, and 520 nm with a multimode microplate reader, respectively.

### 2.12. Statistical Analysis

All data are expressed as means ± SEM and the GraphPad Prism, version6.0 statistical software (GraphPad Software, San Diego, CA, USA) was used for data analysis. A two-tailed Student's *t*-test was used to analyze the significance between two different groups. *P* < 0.05 was considered at statistically significant. Experiments were conducted at least three independent experiments.

## 3. Results

### 3.1. PHL Protects against Cytotoxicity Caused by H_2_O_2_ in DF-1 Cells

**Figure 1:**
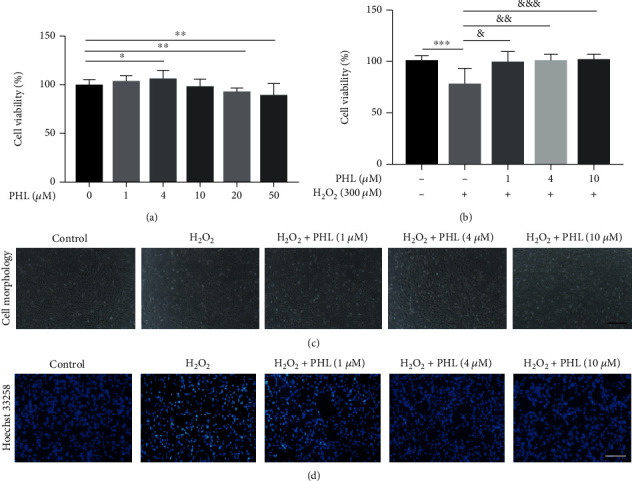
PHL alleviates H_2_O_2_-caused cytotoxicity in DF-1 cells. (a) Cell viability of DF-1 cells treated with PHL at various concentrations (0, 1, 4, 10, 20, and 50 *μ*M) were detected by CCK-8 assay. (b) Cell viability of DF-1 cells treated with PHL at various concentration (1, 4, and 10 *μ*M) for 12 h and followed by H_2_O_2_ (300 *μ*M) for another 3 h were measured by CCK-8 assay. (c) Cell morphology with different treatments were observed by light microscope (Scale bar = 100 *μ*m). (d) Hoechst 33258 staining of each group in DF-1 cells (Scale bar = 100 *μ*m). ^∗^*P* < 0.05, ^∗∗^*P* < 0.01, ^∗∗∗^*P* < 0.001 vs. control group; ^&^*P* < 0.05, ^&&^*P* < 0.01, ^&&&^*P* < 0.001 vs. H_2_O_2_-treated group.

We treated DF-1 cells with different concentration of PHL (0, 1, 4, 10, 20, and 50 *μ*M) for 12 h, and the results showed that PHL at concentration exceeding 10 *μ*M could significantly reduce cell viability (Figure 1(a)). Thus, we chose 1, 4, and 10 *μ*M PHL in the following experiments. Next, we treated cells with PHL (1, 4, and 10 *μ*M) for 12 h and followed by H_2_O_2_ (300 *μ*M) for another 3 h, the results showed that H_2_O_2_ treatment at a concentration of 300 *μ*M significantly reduced the cell viability, whereas PHL pretreatment significantly increased the cell viability in a concentration-dependent manner when compared with H_2_O_2_ treatment alone (Figure 1(b)). Consistently, light microscopy showed that 300 *μ*M H_2_O_2_ treatment markedly inhibited cell proliferation and the most of treated cells were round. In the PHL pretreatment group, the change in the DF-1 cell morphology was improved in a dose-dependent manner, particularly high concentration (10 *μ*M), could significantly promote cell proliferation and adherence (Figure 1(c)). In addition, Hoechst 33258 staining revealed the nuclear fragmentation and apoptotic body increased in 300 *μ*M H_2_O_2_-treated cells, whereas PHL reduced the H_2_O_2_-induced cell apoptosis (Figure 1(d)).

### 3.2. PHL Suppresses H_2_O_2_-Induced Apoptosis in DF-1 Cells

**Figure 2:**
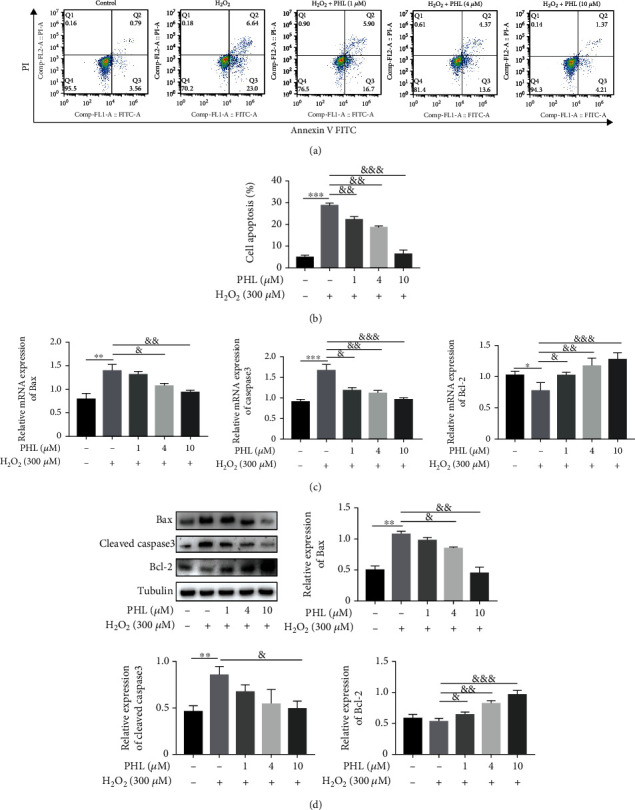
Protective effect of PHL against H_2_O_2_-induced apoptosis in DF-1 cells. (a) Cells were pretreated with different concentrations of PHL for 12 h followed by treatment with 300 *μ*M H_2_O_2_ for another 3 h, apoptosis rates were determined by flow cytometry following Annexin V-FITC/PI staining. (b) Statistical results of apoptosis rate (Q2 + Q3) in each group. (c) The expression of genes involved in apoptosis were detected by qRT-PCR. (d) The expression of proteins involved in apoptosis in DF-1 cells cotreated with PHL and H_2_O_2_ were detected using western blotting. The protein levels of Bax, Cleaved caspase3, and Bcl-2 were quantified from at least three independent experiments in DF-1cells of each group. ^∗^*P* < 0.05, ^∗∗^*P* < 0.01, ^∗∗∗^*P* < 0.001 vs. control group; ^&^*P* < 0.05, ^&&^*P* < 0.01, ^&&&^*P* < 0.001 vs. H_2_O_2_-treated group.

To further determine the effect of PHL on H_2_O_2_-induced apoptosis, we detected the apoptotic rate by flow cytometry analysis. The results showed that 300 *μ*M H_2_O_2_ treatment increased apoptotic rate to 28.7% compared with that in the control group. However, compared to the H_2_O_2_ treatment alone, PHL pretreatment at concentrations of 1, 4, and 10 *μ*M for 12 h prior to H_2_O_2_ treatment significantly decreased the apoptotic rate to 22.2%, 18.5%, and 6.3%, respectively (Figures 2(a)–2(b)). We next examined the gene expression of apoptosis markers such as Bax, Caspase3, and Bcl-2 by qRT-PCR. In H_2_O_2_ alone treated cells, a significant increase in Bax and Caspase3 relative expression and decrease of Bcl-2 relative expression were observed, in contrast, PHL pretreatment effectively against H_2_O_2_-induced apoptosis in a dose-dependent manner (Figure 2(c)). In addition, we analyzed the protein expressions of Bax, Cleaved caspase3, and Bcl-2 by western blot assay, these results were consistent with qRT-PCR data. As shown in Figure 2(d), H_2_O_2_ treatment significantly increased the expression of Bax and Cleaved caspase3 when compared with the control group. Conversely, a high concentration of PHL (10 *μ*M) pretreatment significantly reduced Bax, and Cleaved caspase3 levels, and PHL pretreatment prior to H_2_O_2_ significantly increased Bcl-2 expression in a dose-dependent manner. These results indicate that PHL effectively decreased H_2_O_2_-induced DF-1 cells apoptosis.

### 3.3. PHL Attenuates H_2_O_2_-Induced Oxidative Stress and Mitochondrial Dysfunction in DF-1 Cells

**Figure 3:**
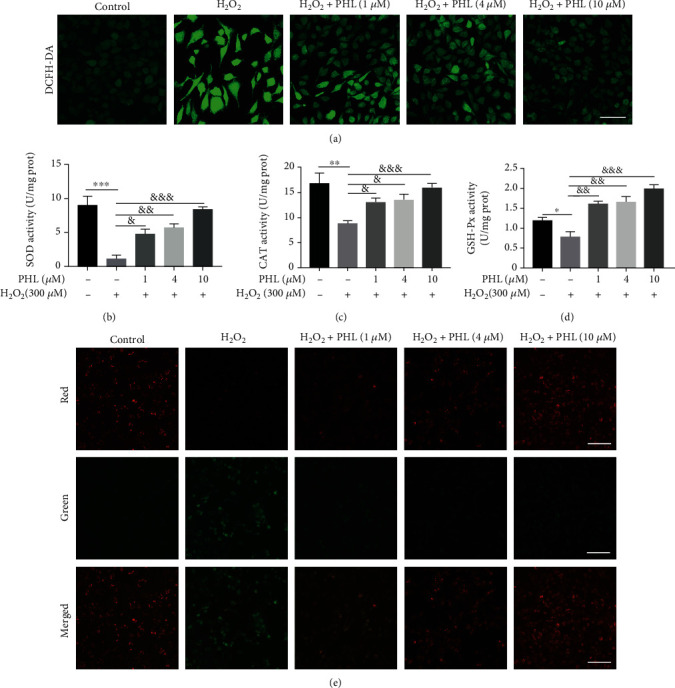
PHL improves abnormal cellular redox state and mitochondrial dysfunction in H_2_O_2_-induced DF-1 cells. (a) Cells were pretreated with different concentrations of PHL for 12 h followed by treatment with 300 *μ*M H_2_O_2_ for another 3 h, ROS levels were detected under a confocal laser-scanning microscopy with a 60× objective following DCFH-DA staining (Scale bar = 50 *μ*m). (b) SOD activity of each group in DF-1 cells. (c) CAT activity of each group in DF-1 cells. (d) GSH-Px activity of each group in DF-1 cells. (e) Cell mitochondrial membrane potential of each group were detected by JC-1 staining under fluorescence microscope (Scale bar = 50 *μ*m). ^∗^*P* < 0.05, ^∗∗^*P* < 0.01, ^∗∗∗^*P* < 0.001 vs. control group; ^&^*P* < 0.05, ^&&^*P* < 0.01, ^&&&^*P* < 0.001 vs. H_2_O_2_-treated group.

Oxidative stress activation and mitochondrial dysfunction are involved in inducing apoptosis. We first investigated the effect of PHL on H_2_O_2_-induced oxidative injury in DF-1 cells by detecting ROS level, SOD, CAT, and GSH-Px activities. DCFH-DA fluorescent probe has been used to indicate intracellular ROS production. We found that ROS level was significantly upregulated in H_2_O_2_-treated DF-1 cells when compared with the control group (Figure 3(a)). And the SOD, CAT, and GSH-Px activities in H_2_O_2_-treated DF-1 cells were lower than controls (Figures 3(b)–3(d)). In comparison, pretreatment with PHL significantly decreased ROS production in cells treated with H_2_O_2_ in a dose-dependent manner (Figure 3(a)). In addition, significant reduced antioxidant enzymes in H_2_O_2_-treated DF-1 cells were counteracted by PHL pretreatment, especially high concentration (Figures 3(b)–3(d)). We next used JC-1 fluorescent probe to assess mitochondrial injury in DF-1 cells with or without PHL pretreatment. As shown in Figure 3(e), the weak green fluorescence and strong red fluorescence was observed in the control group, indicating normal mitochondrial with higher MMP. Compared with control group, the green fluorescence was increased and red fluorescence was decreased after H_2_O_2_ treatment. In contrast, pretreatment with PHL markedly enhanced red fluorescence and weakened green fluorescence in a dose-dependent manner. These data indicate that PHL pretreatment alleviated oxidative stress and mitochondrial injury in H_2_O_2_-treated DF-1 cells.

### 3.4. PHL Promotes Autophagic Flux in H_2_O_2_-Induced DF-1 Cells through the Improvement of Lysosomal Function

**Figure 4:**
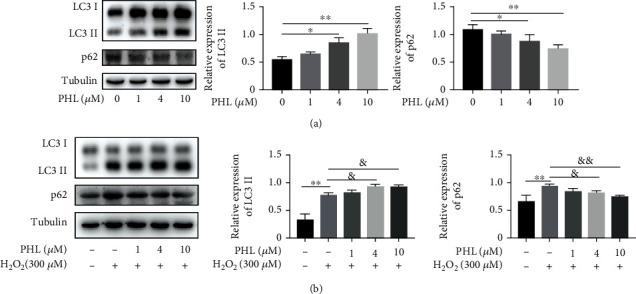
Effect of PHL on autophagy in DF-1 cells with H_2_O_2_ treatment. (a) Cells were treated with different concentration of PHL for 12 h, LC3, and p62 levels were detected by western blotting. Quantitative analysis of LC3-II and p62 protein levels from at least three independent experiments. (b) Cells were pretreated with different concentrations of PHL for 12 h followed by treatment with 300 *μ*M H_2_O_2_ for another 3 h, western blot analysis of LC3 and p62 protein levels, and quantified from at least three independent experiments. ^∗^*P* < 0.05, ^∗∗^*P* < 0.01, ^∗∗∗^*P* < 0.001 vs. control group; ^&^*P* < 0.05, ^&&^*P* < 0.01, ^&&&^*P* < 0.001 vs. H_2_O_2_-treated group.

Previous studies have suggested that inhibition of autophagy could increase intracellular ROS production [26]. To clear the effect of PHL on autophagy, we detected the expression of LC3 and p62, which have been regard as autophagy marker proteins. As shown in Figure 4(a), we observed that LC3-II level increased and p62 level decreased in a dose-dependent manner in PHL-treated DF-1 cells, indicating that PHL induced autophagy. Subsequently, we tested the role of PHL on autophagy in H_2_O_2_-treated DF-1 cells. Compared with the control group, H_2_O_2_ treatment significantly increased LC3-II and p62 protein levels, suggesting the blockage of autophagic degradation. Pretreatment of PHL prior to H_2_O_2_ further increased LC3-II level, whereas the protein expression of p62 reduced in a dose-dependent manner (Figure 4(b)), revealing PHL could promote p62 turnover, that is, promote autophagy.

**Figure 5:**
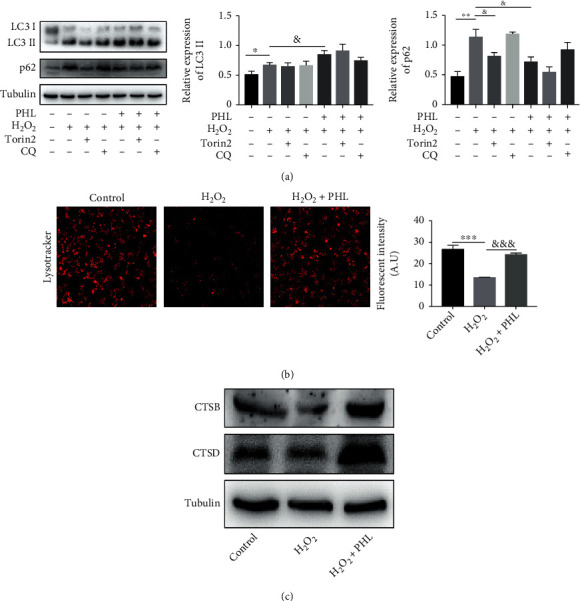
PHL ameliorates H_2_O_2_-blocked autophagy flux and lysosomal dysfunction in DF-1 cells. (a) Cells were cotreated with H_2_O_2_ plus autophagy activator Torin2 or autophagy inhibitor CQ, and PHL pretreatment followed by H_2_O_2_ plus Torin2 or CQ, LC3, and p62 levels were detected by western blotting and quantified from at least three independent experiments. (b) LysoTracker Red staining to evaluate lysosomal pH under a confocal laser-scanning microscopy and quantification of relative fluorescent intensity. (c) Western blot analysis for cathepsin D (CTSD) and cathepsin B (CTSB) protein expressions in DF-1 cells treated with H_2_O_2_ alone and 10 *μ*M PHL pre-treatment followed by H_2_O_2_. ^∗^*P* < 0.05, ^∗∗^*P* < 0.01, ^∗∗∗^*P* < 0.001 vs. control group; ^&^*P* < 0.05, ^&&^*P* < 0.01, ^&&&^*P* < 0.001 vs. H_2_O_2_-treated group.

To get more evidence, we further examined the LC3-II and p62 protein levels in response to H_2_O_2_, H_2_O_2_ + Torin2, H_2_O_2_ + CQ, H_2_O_2_ + PHL, and H_2_O_2_ + PHL + Torin2 and H_2_O_2_ + PHL + CQ treatment. As shown in Figure 5(a), in contrast with the H_2_O_2_-treated group, although Torin2 or CQ cotreatment with H_2_O_2_ did not cause an increase in LC3-II level, the level of p62 was significantly decreased in Torin2 cotreatment with H_2_O_2_ groups, conversely, CQ cotreatment with H_2_O_2_ enhanced the accumulation of p62 level. Compared with H_2_O_2_ + PHL treatment, Torin2 cotreatment with H_2_O_2_ and PHL further promoted p62 turnover, whereas CQ co-treatment with H_2_O_2_ and PHL blocked the degradation of p62, further revealing that PHL could promote the blocked autophagy caused by H_2_O_2_.

Lysosomes play an important role in degrading entrapped components in the autolysosomes [27]. Next, we determined the lysosomal function by LysoTracker Red staining and lysosomal proteases detection. The results showed that the LysoTracker Red puncta decreased and lysosomal pH elevated in H_2_O_2_-treated DF-1 cells. In contrast, PHL pretreatment could remain the acid environment of lysosomes (Figure 5(b)). The expression of cathepsin B (CTSB) and cathepsin D (CTSD), two representative proteases in lysosomes, were decreased in H_2_O_2_-treated DF-1 cells, whereas PHL pretreatment upregulated CTSD and CTSB protein levels (Figure 5(c)). Taken together, these data demonstrate that PHL rescued impairment of autophagic flux in H_2_O_2_-treated DF-1 cells by improving lysosomal function.

### 3.5. PHL Ameliorates H_2_O_2_-Induced Apoptosis via Boosting Autophagic Flux

**Figure 6:**
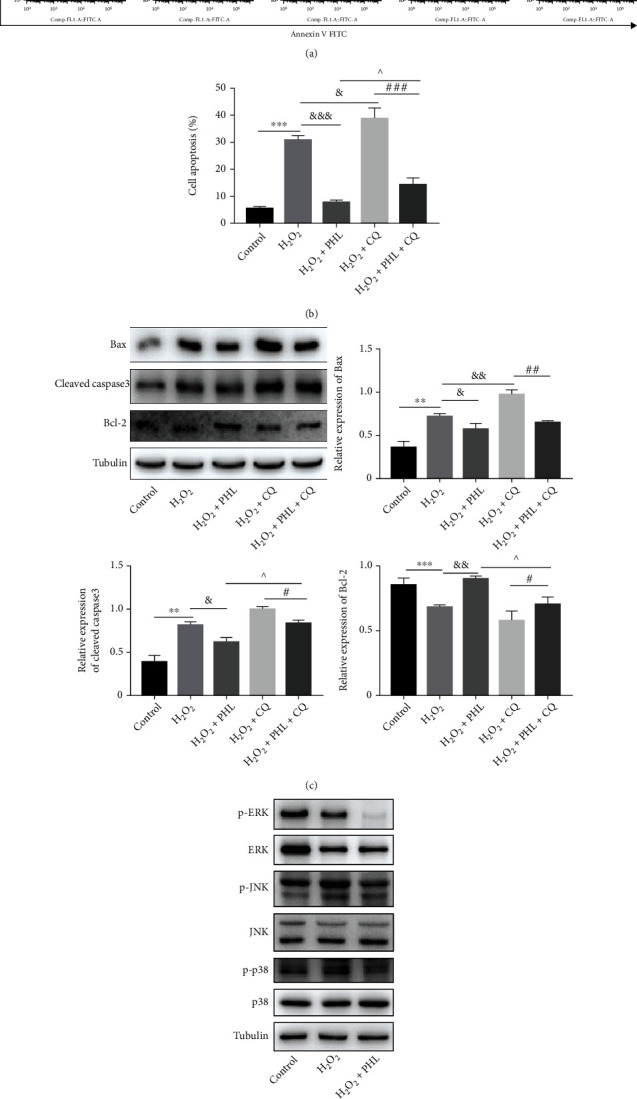
PHL reduces H_2_O_2_-induced apoptosis by promoting autophagy flux. (a) Cells were pretreated with H_2_O_2_ and PHL or CQ together, and apoptosis rate was detected by flow cytometry. (b) Quantification of apoptosis rate of each group (*Q*2 + *Q*3). (c) Western blot analysis for Bax, Cleaved caspase3, and Bcl-2 protein expression in different groups. Quantification analysis of Bax, Cleaved caspase3, and Bcl-2 protein levels from at least three independent experiments. (d) Western blot analysis for p-ERK, ERK, p-JNK, JNK, p-p38, and p38 protein levels. ^∗^*P* < 0.05, ^∗∗^*P* < 0.01, ^∗∗∗^*P* < 0.001 vs. control group; ^&^*P* < 0.05, ^&&^*P* < 0.01, ^&&&^*P* < 0.001 vs. H_2_O_2_-treated group.

We next sought to determine whether the effect of PHL against H_2_O_2_-induced apoptosis depended on autophagic flux activation. We used autophagy inhibitor CQ that block autophagic flux, the results showed that compared with H_2_O_2_ treatment alone, PHL pretreatment reduced apoptosis rate, whereas CQ cotreatment caused higher apoptosis. In contrast with H_2_O_2_ and PHL cotreatment, the effect of PHL was counteracted by CQ. Consistently, PHL effectively repressed the H_2_O_2_ + CQ-induced increase in apoptosis rate (Figures 6(a)–6(b)). Meanwhile, higher protein levels of Bax and Cleaved caspase3, and lower Bcl-2 level were observed in H_2_O_2_ and CQ cotreated group that compared with H_2_O_2_ treatment alone, but this effect was counteracted by PHL pretreatment. Also, CQ repressed Bcl-2 protein expression and increased Bax and Cleaved caspase3 protein expressions that have been improved in H_2_O_2_ and PHL cotreated group (Figure 6(c)). Moreover, H_2_O_2_ treatment promoted the phosphorylation of ERK, JNK, and p38 when compared with the control group, whereas PHL pretreatment suppressed the activation of ERK, JNK, and p38 MAPK signal pathways in H_2_O_2_-treated DF-1 cells (Figure 6(d)). These results suggest that PHL reduced H_2_O_2_-induced oxidative damage and promoted autophagic flux may be related to the suppression of MAPK signaling pathways in DF-1 cells.

## 4. Discussion

The present study demonstrates that PHL can against H_2_O_2_-induced oxidative injury and apoptosis in DF-1 cells. Specifically, PHL increases MMP, reduces ROS production, improves redox balance, maintains lysosomal stability, and restores autophagic flux, as well as PHL suppresses the levels of ERK, JNK, and p38 phosphorylation in the H_2_O_2_-treated DF-1 cells. Obviously, our results provided evidence that PHL might act as a reasonable plant derived substances for stress defense in poultry production.

PHL is a natural flavonoid compound present in the peel and root bark of apples, strawberries, and other plants. Numerous studies have demonstrated its beneficial role in combating oxidative stress [13, 16, 28]. In this study, we first determined the nontoxic effect concentration of PHL in DF-1 cells. The cell viability assay result showed that PHL had no toxic effects on cell viability when the concentration is lower than 20 *μ*M, and consistent results were obtained in other cell models [14, 28, 29]. Therefore, PHL at the concentration of 1, 4, and 10 *μ*M were selected for subsequent experiments. Next, DF-1 cells were treated with 300 *μ*M H_2_O_2_ to establish an oxidative stress model [30]. We observed that the cell viability was significantly reduced when stimulated to 300 *μ*M H_2_O_2_, and cells showed round morphology and the nuclei were shriveled. While PHL pretreatment significantly increased the decrease of cell viability caused by H_2_O_2_ in a dose-dependent manner, and promoted cell proliferation as well as adherence. Oxidative stress can cause cell apoptosis [31]. In this study, H_2_O_2_ increased apoptotic nuclei as demonstrated via Hoechst 33258 staining when compared with control cells. Similar results were confirmed using flow cytometry. The antiapoptotic member of Bcl-2 and proapoptotic member of Bax are major proteins used to indicate apoptosis [32]. We also detected the expressions of apoptosis-related genes and proteins, and found that H_2_O_2_-treated DF-1 cells showed increased Bax and Cleaved caspase3 levels, and decreased Bcl-2 level, suggesting oxidative stress indeed promote cell apoptosis. In contrast, PHL pretreatment obviously reduced the number of apoptotic nuclei in H_2_O_2_-treated DF-1 cells, and significantly reduced the apoptotic rates. Furthermore, the increased expression of Bax and Cleaved caspase3 and the reduced expression of Bcl-2 in H_2_O_2_-treated cells were reversed by PHL pretreatment in a dose-dependent manner. Overall, our results demonstrate that PHL exhibits a protective effect from H_2_O_2_-induced damage and apoptosis in DF-1 cells.

ROS is considered as the main factor regulating oxidative stress. Under physiological conditions, the production of ROS is in balance with the elimination of endogenous antioxidant systems [33]. H_2_O_2_ leads to overproduction of ROS, which can trigger apoptosis in various tissues and cells [34]. We observed a significant increase in ROS level in H_2_O_2_-treated DF-1 cells. Instead, PHL pretreatment obviously reduced ROS generation induced by H_2_O_2_. Consistent with our results, several studies have reported that PHL could reduce ROS accumulation in models of oxidative stress induced by other substances [35, 36]. SOD, CAT, and GSH-Px are major ROS scavengers that reduce oxidative stress. PHL has been confirmed to exert antioxidant activity by activating antioxidant enzymes [16]. In the present study, PHL pretreatment significantly improved SOD, CAT, and GSH-Px activities in a dose-dependent manner in H_2_O_2_-treated cells, suggesting that PHL has the ability in maintaining redox balance. Damaged mitochondrial induced by oxidative stress can produce more ROS which further aggravated mitochondrial injury [37] and lead to mitochondrial membrane depolarization [38]. Our results showed that PHL attenuated H_2_O_2_-induced decrease in MMP, as demonstrated by the increased ratio of red (JC-1 aggregates)/green (JC-1 monomers) fluorescence intensity when compared with H_2_O_2_-treated group. The above results indicate that PHL could effectively improve redox status homeostasis and mitochondrial function.

Autophagy is a catabolic process that protects cell from various stressors [39]. The role of autophagy in oxidative stress injury is still controversial. Autophagy has been reported to play a positive effect in promoting cell survival and antiapoptosis under H_2_O_2_-induced oxidative stress [40, 41]. However, other studies have shown that H_2_O_2_ induces cytotoxicity by triggering autophagy [42, 43]. ROS activates autophagy as an intracellular key factor to clear damaged mitochondrial at the early stage of oxidative stress, but excessive and dysregulated autophagy caused by the acute and continuous stimulation fails to remove cytoplasmic damaged organelles, leading to ROS overproduction and oxidative stress, aggravating the organelles damage, and eventually promoting cell death [44, 45]. LC3 and p62 are key marker proteins of autophagy. LC3 is involved in autophagosome formation, and p62 is involved in the formation and degradation of aggregation proteins [46]. Our results showed a significant increase in LC3-II and p62 levels in H_2_O_2_-treated group when compared with the control group, indicating the autophagic flux was blocked. We speculated that the difference in this result may be related to the cell type and the concentration and time of H_2_O_2_ stimulation. Different cells have different sensitivity to H_2_O_2_ stimulation. Previous studies have reported that PHL could enhance autophagy and promote autophagic flux [17, 47], and our data confirmed this result. In the present study, compared with the control group, PHL significantly increased LC3-II protein expression at concentrations exceeding 1 *μ*M. On the contrary, the protein level of p62 was found markedly declined in PHL treated DF-1 cells, suggesting the increase in autophagic flux after PHL treatment. Subsequently, we investigated the effect of PHL on autophagy in H_2_O_2_-treated cells. PHL pretreatment further increased LC3-II protein expressions, but promoted p62 degradation in a dose-dependent manner, suggesting PHL effectively reversed the blocked autophagic flux in the H_2_O_2_-treated cells. In addition, we used the autophagy activator Torin2 and autophagy inhibitor CQ to further evaluate the effect of PHL on autophagic flux in H_2_O_2_-treated cells. We found that the increased LC3-II and decreased p62 levels in the Torin2 and H_2_O_2_ cotreated group is like that of PHL and H_2_O_2_ cotreatment, that is, PHL and Torin2 are similar in promoting autophagic flux. Moreover, the increased LC3-II accumulation and reduced p62 degradation upon Torin2 and PHL cotreatment in H_2_O_2_-treated cells were observed when compared with Torin2 or PHL alone. Conversely, CQ could counteract the role of PHL for promoting autophagic flux.

Autophagy is dynamic intracellular degradation process, phagosomes form mature autophagosomes and fuse with lysosomes to form a degradative autolysosome, and this complete process is termed autophagic flux [48]. Mechanistically, the impaired lysosomal function contributes to blocking autophagic flux [49]. We further investigated the effect of PHL on lysosomal function in H_2_O_2_-treated DF-1 cells. The lysotracker red probe result showed that H_2_O_2_ significantly decreased the red fluorescence intensity, suggesting H_2_O_2_ destroyed the acidic environment of lysosomes. On the contrary, compared with the H_2_O_2_ treatment alone, PHL pretreatment significantly elevated the red fluorescence intensity. Moreover, the expression of CTSB and CTSD proteins, two major lysosomal proteases responsible for autophagy degradation, were markedly reduced in H_2_O_2_-treated cells, which were reversed by PHL pretreatment. Collectively, PHL could promote autophagic flux by maintaining lysosomal acidic environment and enhancing cathepsin proteases activities.

To test whether PHL-enhanced autophagic flux decreased H_2_O_2_-induced cell apoptosis, DF-1 cells were treated with or without PHL and/or CQ before H_2_O_2_ treatment. Our results showed that CQ further increased H_2_O_2_-induced cell apoptosis, evidenced by upregulation of Bax, Cleaved caspase3 expression, and apoptosis rates as well as downregulation of Bcl-2 protein expression, which was reversed by PHL. These results indicate that PHL alleviated H_2_O_2_-induced cell apoptosis by enhancing autophagic flux.

Previous researches have shown that MAPK signaling pathway including p38, JNK, and ERK plays a key role in cell apoptosis and autophagy [50]. We further examined the change in three subunits of MAPK pathway, our study found that the phosphorylation levels of ERK, JNK, and p38 were upregulated in H_2_O_2_-treated group, suggesting activated MAPK signaling pathway, consistence with other findings that oxidative damage or stress leads to MAPKs activation [51–53]. In contrast, pretreatment with PHL could effectively inhibit MAPKs subunits phosphorylation levels. Thus, we speculated that the protective effect of PHL is at least partially dependent on the MAPKs signaling pathways, but further studies are needed to verify the exact role of MAPKs.

At last, although PHL has a variety of biological activities, its application as a drug and additive has been restricted due to its low aqueous solubility, poor absorption and bioavailability, and PHL is quickly excreted from the body after absorption [54]. At present, the problem of poor absorption and bioavailability of PHL has been solved by changing its dosage form such as self-nano emulsion [55], liposome [56], and microemulsion formulation [57]. However, PHL is found in very small amounts in organic matter, it is necessary to develop chemical synthesis processes that can meet further needs to develop more PHL synthetic/semisynthetic derivatives and to improve the efficiency of plant extract at target parts in the body. In addition, despite the existing data, we believe that further research is needed on PHL. We need to verify the role of PHL in the oxidative stress model of poultry in vivo, and the exact molecular mechanism of phloretin's biological role needs to be further clarified. Furthermore, given that fruit by-products have other economic value, PHL may be a cost-effective alternative with no positive impact on economic efficiency compared to supplements currently used in the poultry industry. Therefore, more research is also needed on the effects of fruit by-products and their related extracts to find a cheaper alternative to antibiotics.

## 5. Conclusion

In conclusion, this is the first study on the protective effect and potential mechanism of PHL against H_2_O_2_-induced oxidative injury and apoptosis in DF-1 cells. We draw the conclusion that PHL ameliorates H_2_O_2_-induced DF-1 cell apoptosis by improving lysosomal function and boosting autophagic flux, and the inactivation of MAPKs signaling pathway might be responsible for its protective effect to prevent H_2_O_2_-induced DF-1 cell injury.

## Data Availability

The data used to support the findings of this study are available from the corresponding author upon logical request.
